# Retraction: Mangiferin, a Natural Xanthone, Protects Murine Liver in Pb(II) Induced Hepatic Damage and Cell Death via MAP Kinase, NF-κB and Mitochondria Dependent Pathways

**DOI:** 10.1371/journal.pone.0362434

**Published:** 2026-11-11

**Authors:** The PLOS One Editors

Following the publication of this article [1], concerns were raised with results published in Figs 6 and 9-10. Specifically,

- There appear to be multiple unusually repetitive clusters within and between the Normal, Pb(II), and Pb(II) & Mang panels in Fig 6.
- The following panels appear more similar than would be expected from independent results:
  - Fig 9 Liver tissue Bax and Fig 10B Liver tissue Cytochrome c.
  - Fig 9 Liver tissue Bcl-2 and Fig 10B Liver tissue Apaf 1.
  - Fig 9 Liver tissue β-actin, Fig 10B Liver tissue Caspase 3, and Fig 10B Liver tissue β-actin.
- The following lanes appear more similar than would be expected from independent results:
  - Fig 9 Hepatocytes β-actin panel, lanes 1 and 2.
  - Fig 9 Hepatocytes β-actin panel, lanes 3 and 4.

The corresponding author stated that they agreed similarities can be observed in the FACS results, but stated that the overall cellular distribution in these panels is different both at micro and macro level. Regarding the similarity between the Fig 9 Liver tissue Bcl-2 and Fig 10B Liver tissue Apaf 1 panels, the corresponding author stated that this was due to a figure preparation error. They stated that they disagree with the journal’s other observations involving Figs 9 and 10, and stated that they are unable to retrieve the original raw data files. In the absence of the underlying data, these concerns cannot be resolved.

In light of the above concerns, which call into question the reliability and integrity of the published results, the *PLOS One* Editors retract this article.

KS agreed with the retraction. PCS did not agree with the retraction. PBP responded but expressed neither agreement nor disagreement with the editorial decision.

In addition:

- The Fig 9 Liver tissue Bax and Fig 10B Liver tissue Cytochrome c panels in [1] appear similar to the Fig 2C MCP-1 panel in [2] when flipped, which was published later by a different author group.
- The Fig 10B Hepatocytes β-actin panel in [1] appears similar to the Fig 5F Cleaved caspase 9 panel in [3] when flipped, which was published later by a different author group.

## References

[1] Pal PB, Sinha K, Sil PC. RETRACTED: Mangiferin, a natural xanthone, protects murine liver in Pb(II) induced hepatic damage and cell death via MAP kinase, NF-κB and mitochondria dependent pathways. PLoS One. 2013;8(2):e56894. doi: 10.1371/journal.pone.0056894 23451106 PMC3581562

[2] Wang W, Yang B, Cui Y, Zhan Y. Isoliquiritigenin attenuates spinal tuberculosis through inhibiting immune response in a New Zealand white rabbit model. Korean J Physiol Pharmacol. 2018;22(4):369–77. doi: 10.4196/kjpp.2018.22.4.369 29962851 PMC6019872

[3] Dua TK, Dewanjee S, Khanra R, Bhattacharya N, Bhaskar B, Zia-Ul-Haq M, et al. The effects of two common edible herbs, *Ipomoea aquatica* and *Enhydra fluctuans*, on cadmium-induced pathophysiology: a focus on oxidative defence and anti-apoptotic mechanism. J Transl Med. 2015;13:245. doi: 10.1186/s12967-015-0598-6 26215156 PMC4515882

